# Relationship between bite force, occlusal contact area, and three-dimensional facial soft tissue in dentofacial deformities

**DOI:** 10.1590/2317-1782/20242023203en

**Published:** 2024-04-29

**Authors:** Joana Carolina Martins Simões, Denny Marcos Garcia, Francisco Veríssimo De Mello-Filho, Claudia Maria De Felício, Luciana Vitaliano Voi Trawitzki

**Affiliations:** 1 Departamento de Oftalmologia, Otorrinolaringologia e Cirurgia de Cabeça e Pescoço, Faculdade de Medicina de Ribeirão Preto – FMRP, Universidade de São Paulo – USP - Ribeirão Preto (SP), Brasil.; 2 Núcleo de Apoio à Pesquisa em Morfofisiologia Craniofacial – NAP-CF, Faculdade de Medicina de Ribeirão Preto – FMRP, Universidade de São Paulo – USP - Ribeirão Preto (SP), Brasil.

**Keywords:** Dentofacial Deformities, Orthognathic Surgery, Three-dimensional Imaging, Bite Force, Mastication, Stomatognathic System, Myofunctional Therapy

## Abstract

**Purpose:**

This study aimed to investigate three-dimensional facial soft tissue dimensions, maximum bite force (MBF), and occlusal contact area in patients with DFD. In addition, we analyzed the relationship between MBF and the three-dimensional facial measurements.

**Methods:**

Thirty-two patients with skeletal Class III DFD and 20 patients with Class II DFD underwent a soft tissue evaluation using surface laser scanning, as well as MBF and occlusal contact area assessments. The DFD groups were compared with each other and with 25 healthy subjects.

**Results:**

Significant morphological differences were found in the transversal, vertical, and anteroposterior dimensions between Class II DFD and Class III DFD. Both DFD groups presented an increased linear distance of chin height, which was strongly related with decreased MBF magnitude. The DFD groups exhibited lower MBF and occlusal contact area, with no significant differences between Class II and Class III DFD.

**Conclusion:**

The presence of DFD affected 3D measurements of facial soft tissue, causing variations beyond normal limits, lower MBF, and occlusal contact area in both Class II and Class III DFD patients. The vertical dimension might have influenced the lower MBF magnitude in the studied skeletal deformities.

## INTRODUCTION

Dentofacial deformities (DFD) are characterized by complex three-dimensional (3D) skeletal abnormalities of the mandible, maxilla, or both. There is a direct relationship between facial skeletal abnormalities, malocclusion, and the masticatory function. Therefore, patients with Class II and Class III DFD have functional impairments, such as abnormal chewing patterns, swallowing difficulties, and speech disorders^(1)^. The treatment involves orthognathic surgery, and the primary purpose is to improve the facial aesthetic appearance and the masticatory function, including bite force and occlusal contact area^(2,3)^.

Bite force is a component of the masticatory function^(2)^ and an effective indicator of the capacity of the mandibular muscles^(3)^. It has been reported that there is a reduction in maximum bite force (MBF) values in individuals with Class II and Class III DFD compared to healthy subjects^(4)^. Despite the morphological discrepancies, there are no differences in MBF between these deformities^(4,5)^. However, the studies did not evaluate the soft tissue dimensions, which are also essential for the performance of the stomatognathic functions and esthetic concern.

There is a connection between soft and hard tissues^(6,7)^. Vertical craniofacial growth patterns reduced the size of the jaw muscles^(8)^ and the magnitude of MBF^(9)^. We believe there is a relationship between masticatory components and facial morphology also in DFD context. Although there have been few reports in Class II and Class III DFD patients. The studies mostly used the two-dimensional (2D) approach and focused on improvements after orthognathic surgery^(10,11)^. The laser scanning technique has been applied in DFD for assessing the facial soft tissue, overcoming the limitations of 2D devices in the 3D structure, and improving the diagnosis and treatment planning^(7,12,13)^.

Therefore, clarifying the relationship between soft structures and masticatory function in patients with Class II and Class III DFD before the surgical changes in the face is clinically significant. Frequently the therapeutic approach will differ according to the type of DFD. Thus, it is relevant to know the morphofunctional characteristics even in the diagnosis, as it may impact postoperative myofunctional management. This knowledge can help an individualized and successful therapeutic planning.

This study aimed to evaluate 3D facial soft tissue dimensions, MBF, and occlusal contact area in patients with Class II and Class III DFD before orthognathic surgery, as well as in healthy subjects. In addition, the study aimed to investigate the relationship between MBF and the 3D facial measurements to analyze the masticatory function in DFD patients.

## METHODS

### Subject selection

This cross-sectional observational study was approved by the Institutional Research Ethics Committee (process number 31582114.0.0000.5440), following the principles of the Helsinki Declaration. All subjects provided written informed consent to participate.

The study involved 77 patients with DFD, including 32 with skeletal Class III DFD (group DFD III, 15 men and 17 women, mean age ± standard deviation = 27 ± 7 years, ranging from 18 to 40 years old) and 20 with skeletal Class II DFD (group DFD II, 4 men and 16 women, 26 ± 6 years, ranging from 18 to 36 years old). The diagnosis was based on cephalometric analyses, photographs, plaster models, and clinical evaluations by orthodontists and surgeons at a university public hospital, where several complex treatments have been executed through the Unified Health System (SUS). Orthosurgical planning was performed by the same team, based on a convenience demand, which is often mostly composed of patients with Class III DFD. Jaw relationship and skeletal malocclusion were characterized by measuring the angles SNA, SNB and ANB. The DFD patients were undergoing preparatory orthodontic treatment for orthognathic surgery. The control group (CG) consisted of 25 healthy subjects (9 men and 16 women, 24 ± 4 years, ranging from 18 to 31 years old).

The inclusion criteria for the DFD groups were the presence of skeletal Class II malocclusion (mandibular retrognathism, excessive maxillary growth, or both) or skeletal Class III malocclusion (mandibular prognathism, maxillary deficiency, or both). The inclusion criteria for the CG were good general health, thirds of the face in normal balance, Angle Class I molar relationship, at least 28 teeth, and overjet and overbite between 2 mm and 4 mm.

The exclusion criteria for all groups were the presence of condylar hyperplasia, trauma, tumor, or surgery in the head and neck regions; central or peripheral neurological disorders; chronic use of analgesic, anti-inflammatory, or psychotropic drugs; and current or prior orthodontic or orofacial myofunctional treatment. Subjects lacking more than one tooth on either side of the upper and lower arch (except the third molars) were also excluded. Subjects showing signs and symptoms of temporomandibular disorder and orofacial myofunctional disorder were also excluded from the CG.

### Interview and clinical examination

All participants were interviewed to gather personal information and assess their eligibility based on the study’s inclusion and exclusion criteria. The orofacial myofunctional condition was evaluated using the Protocol for Orofacial Myofunctional Evaluation with Scores (OMES)^(14)^. This protocol accurately indicates the presence or absence of orofacial myofunctional disorder or any alterations that may affect the measurement of bite force. The presence or absence of signs and symptoms of temporomandibular disorder was assessed using the Protocol for Multi-Professional Centers for the Determination of Signs and Symptoms of Temporomandibular Disorder (ProTMDmulti-Part II)^(15)^.

All procedures of this study were performed by the same examiner, a Speech-Language Pathologist trained and expert in the area (J.C.M.S). The evaluations were executed approximately one month before the surgical treatment for the DFD groups and recorded preferably during only one day for all study groups. All analyses were made by the same examiner (J.C.M.S) in collaboration with our group’s physicist (D.M.G.).

### Three-dimensional morphology assessment

The laser scanning technique was used to acquire facial morphology data using the FastSCAN^TM^ Scorpion laser scanner system (Polhemus, Inc., Colchester, USA). This high-resolution equipment rapidly scans the surface of an object and provides 3D coordinates for surface points^(16)^. The laser scanning system consists of a laser source projected into the subject’s face and captured by two cameras positioned at different angles. To correct postural movements, a transmitter fitted to a pedestal facing the individual’s back and a receiver sensor attached to the head were used. The scanned surface was then reconstructed in a virtual 3D model on a connected personal computer based on the spatial position of the cameras and the laser deformation on the subject’s face.

The 3D images of the patients were acquired at the final stage of the preparatory orthodontic treatment for orthognathic surgery. During image acquisition, the subjects stood upright with their backs to the pedestal, looking straight ahead and maintaining a natural head position. They were instructed to assume their usual resting (neutral) facial expression with relaxed body musculature. They were not allowed to use adornments, such as earrings, glasses, or caps.

### Landmarks

**Figure 1:**
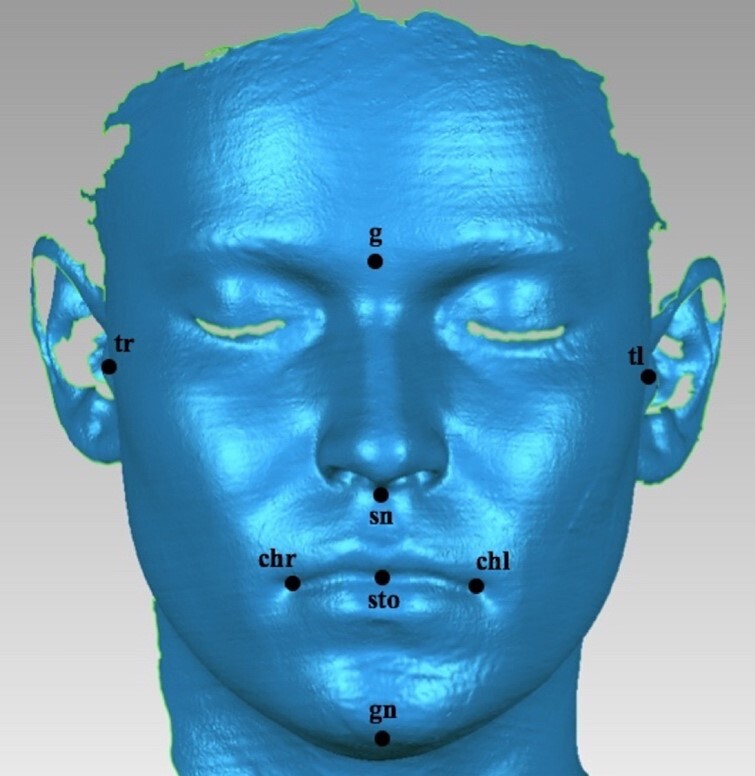
Facial scan representing the anthropometric landmarks used for the surface facial measurements

The software Geomagic Studio (Geomagic, ResearchTriangle Park, NC, USA) was used to identify the anthropometric soft tissue landmarks. The original point (0, 0, 0) was defined as being the reference landmark itself. The coordinates represented by the three axes of the Cartesian coordinate system were applied on each original point and used to calculate a set of 3D soft tissue measurements: y (vertical dimension), a midsagittal line; x (transversal dimension), a perpendicular line to the midsagittal line; and z (anteroposterior dimension), a parallel line to the floor. Each landmark was selected based on its easy location and identification in the 3D virtual image, following the anatomical description proposed by Farkas and Deutsch^(17)^. A total of eight anthropometric landmarks was selected and measured in 3D coordinates *(x,y,z)*: glabella (g); subnasale (sn); right tragion (tr); left tragion (tl); stomion (sto); right cheilion (chr); left cheilion (chl), and gnathion (gn) (Figure 1).

**Caption:** glabella (g), subnasale (sn), right tragion (tr), left tragion (tl), stomion (sto), right cheilion (chr), left cheilion (chl), gnathion (gn)

### Linear and proportional measurements

The 3D measurements were used to calculate the soft tissue facial dimensions, which were based on linear distances between two landmarks measured in millimeters (mm) and calculated using Equation 1.


$$d_{A.B}=\sqrt{{\left(x_{B}-x_{A}\right)}^{2}+{\left(y_{B}-y_{A}\right)}^{2}+{\left(z_{B}-z_{A}\right)}^{2}}$$ (1)

The following indices were derived from the *x, y, z* coordinates: total facial height (g-gn); upper facial height (g-sn); lower facial height (sn-gn); lip height (sn-sto); chin height (sto-gn); right side (tr-chr); left side (tl-chl); middle facial depth (t-sn); lower facial depth (t-gn), where t represents the midpoint between right tragion and left tragion; and middle face width (tr-tl). Facial proportional measurements were determined using the ratio between two distances, multiplied by 100 (percentage ratio): facial height/facial width (g-gn/tr-tl); upper facial height/lower facial height (g-sn/sn-gn); middle facial depth/lower facial depth (t-sn/t-gn); lip height/chin height (sn-sto/sto-gn); and the asymmetry between the right and left sides (tr-chr/tl-chl).

The 3D coordinates of linear and proportional measurements for facial morphology were calculated, and the data were transferred to Microsoft Excel (version 16.30).

### Recording of maximum bite force

The MBF was measured using an electronic gnathodynamometer (DDK/M, Kratos® Equipamentos Industriais, Cotia, São Paulo, Brazil). This device, specifically designed for determining MBF in humans, has capacity of 980 Newtons (N). It was manufactured in an aluminum box, with a 10 mm load cell, a 5-digit display for reading, and a push-button mechanism that allows precise control of the obtained values. It also has a maximum peak memory and scales for N, kilogram-force (Kgf), and pounds-force (Ibf). To adapt the equipment to oral conditions, two rods containing two teflon discs were used, which were coated with disposable latex finger cots (Mucambo S.A., Bahia, Brazil) for each examination. The gnathodynamometer demonstrated good repeatability, with a technical error of measurements (TEM) = 70.00, interclass correlation coefficient test-retest intra-rater reliability = 0.99, and inter-rater reliability = 0.96^(18)^.

During the recording of MBF, the subjects were instructed to seat comfortably in a chair without head support, with their feet resting on the floor, and their shoulders and arms in a relaxed position. The gnathodynamometer was placed between the maxillary and mandibular first molar teeth, alternating between the right and left sides. The subjects were instructed to bite as forcefully as possible three times, with a 2-min rest interval between recordings^(4)^. The average force on the right and left sides was used for analysis.

### Recording of occlusal contact area

The T-Scan 8 (Tekscan Inc., South Boston, MA, USA) was used to assess the occlusal contact area. This system consists of a sensor handle and a pressure-sensitive sensor with a thickness of 100 micrometer (μm), connected to a personal computer. It enables the analysis and storage of virtual occlusal contacts. During data recording, the subjects were instructed to sit up in the chair and to bite into maximum intercuspation with the sensor placed between the dental arches. The virtual color image of each subject, represented by numerous sensor elements, was analyzed using a software developed in Matlab version 8.5 (MathWorks, Natick, MA, U.S.A.). Through image processing techniques, the occlusal area of right, left, and both sides could be determined. The occlusal contact asymmetry between sides was calculated using the index proposed by Naeije et al.^(19)^.

### Statistical analysis

The results were presented as mean and standard deviation (SD) or median and interquartile range (IQR) when non-parametric tests were applied. Comparisons between the GC, DFD II, and DFD III groups were performed using analysis of variance (ANOVA) for independent samples. When a statistical difference was found, the Tukey’s honestly significant difference (Tukey-HSD) post hoc test was applied. For variables that did not meet the assumptions for ANOVA (Levene’s test for homogeneity of variance and Shapiro-Wilk test for normality), the Kruskal-Wallis non-parametric test was used as an alternative, followed by Dunn’s post hoc test. Spearman’s correlation test was used to assess the associations between bite force measurements and morphology. Multiple linear regression, with the stepwise selection method, using the Akaike information criterion, was used to identify anthropometric variables related to bite force, with age and sex as covariates.

In this study, effect size was calculated to quantify the magnitude of differences and indicate the clinical relevance of the results, regardless of sample size. Partial eta squared ( $\eta_{p}^{2}$ ) was calculated for ANOVA, and epsilon squared ( $E_{R}^{2}$ ) was calculated for the Kruskal-Wallis test^(20)^. For ANOVA, the cutoff values were $\eta_{p}^{2}$ = 0.0099 for small; $\eta_{p}^{2}$ = 0.0588 for medium; and $\eta_{p}^{2}$ = 0.1379 for large^(21)^. For the Kruskal-Wallis test, the cutoff values were $E_{R}^{2}$ = 0.04 for small; $E_{R}^{2}$ = 0.25 for medium; and $E_{R}^{2}$ = 0.64 for large^(22)^. All statistical analyses were performed using JMP Software (SAS Institute Inc., Cary, NC, USA.), with a significance level of 5% (p < 0.05).

The reliability of the facial scanning method was assessed using the TEM^(23)^, Lin’s concordance correlation coefficient^(24)^, and the paired Student *t*-test to check for systematic error. Validation was conducted in two ways: intra-rater, where measurements were taken by the same rater on different days, and inter-rater, where measurements were taken on the same day by two different raters.

## RESULTS

### Anthropometric measurement reliability

Intra-rater reliability showed a TEM value of 1.08 mm, indicating no systematic error (p = 0.1184), and a Lin’s concordance correlation coefficient of 0.999. Inter-rater analysis revealed a TEM of 1.48 mm, also without systematic error (p = 0.2893), and a Lin’s concordance correlation coefficient of 0.998.

### Surface facial linear distances

Significant inter-group differences (p ≤ 0.05) were observed for all linear distances between the anthropometric landmarks, except for upper facial height (g-sn), as shown in Table 1. Post-hoc tests revealed that the DFD II group had increased vertical measurements of total facial height (g-gn), lower facial height (sn-gn), and chin height (sto-gn) compared to the CG. Additionally, the DFD II group presented a smaller lower facial depth (t-gn). Moreover, the DFD III group showed increased chin height (sto-gn) and lower facial depth (t-gn) distances, as well as a decrease in middle facial depth (t-sn), compared to the CG.

**Table 1:** Linear distances and facial proportions of the three groups

**Measurements**		**CG (n=25)**		**DFD II (n=20)**		**DFD III (n=32)**		**ANOVA P-Value** ^*^		$\eta_{p}^{2}$
**Linear distances (mm)**		**Mean**	**SD**	**Mean**	**SD**	**Mean**	**SD**			
Total facial height (g-gn)		126.6 ^a^	5.4	132.3 ^b^	6.1	129.1 ^a, b^	7.2		0. 0162*	0.107
Middle face width (tr-tl)		145.8 ^a. b^	6.5	140.9 ^a^	8.4	146.9 ^b^	7.6		0. 0206*	0.101
Upper facial height (g-sn)		64.2	3.6	65.4	3.0	63.6	4.5		0. 27	0.035
Lower facial height (sn-gn)		64.1 ^a^	4.2	70.9 ^b^	6.3	66.1 ^a^	5.2		0. 0002*	0.209
Lip height (sn-sto)		20.6 ^a, b^	2.4	24.5 ^a^	12.0	19.4 ^b^	2.8		0. 0227*	0.096
Chin height (sto-gn)		43.7 ^a^	2.5	49.7 ^b^	6.9	47.1 ^b^	3.7		0. 0001*	0.217
Middle facial depth (t-sn)		102.7 ^a^	5.6	98.5 ^a. b^	6.4	96.8 ^b^	7.0		0. 0033*	0.141
Lower facial depth (t-gn)		119.3 ^a^	6.0	110.3 ^b^	6.4	124.4 ^c^	7.2		<. 0001*	0.430
Right side (tr-chr)		106.9 ^a. b^	4.7	103.4 ^a^	6.9	107.5 ^b^	4.7		0. 0263*	0.095
Left side (tl-chl)		106.8 ^a^	4.3	101.1 ^b^	5.2	106.5 ^a^	4.2		<. 0001*	0.232
**Facial proportions (%)**		**Median**	**IQR**	**Median**	**IQR**	**Median**	**IQR**		**Kruskal-Wallis P-Value** ^**^	$E_{R}^{2}$
Facial height/facial width (g-gn/tr-tl)		87.5 ^a^	3.7	94.6 ^b^	8.8	88.9 ^a^	9.7		0. 0001**	0.262
Upper facial height/lower facial height (g-sn/sn-gn)		101.4 ^a^	15.8	92.1 ^b^	12.3	97.1 ^a, b^	12.2		0. 0248**	0.104
Middle facial depth/lower facial depth (t-sn/t-gn)		86.2 ^a^	5.0	89.0 ^a^	4.8	78.1 ^b^	4.6		<. 0001**	0.672
Lip height/chin height (sn-sto/sto-gn)	47.3 ^a^		8.7	46.1 ^a, b^	11.5	42.2 ^b^	11.4		0. 0042^**^	0.161
Symmetry between sides (tr-chr/tl-chl)		1.4	2.2	2.1	3.8	1.4	1.6		0. 16	0.074

Analysis of variance (ANOVA) test*. Mean standard deviations (SD). Superscript letters indicate differences in the Tukey-HSD post-test, and the partial eta squared ( $\eta_{p}^{2}$ ) represents the effect size for the linear distances;

Kruskal-Wallis test**. Median values. Interquartile range (IQR). Superscript letters indicate differences in Dunn’s post-test, and the epsilon squared ( $E_{R}^{2}$ ) represents the effect size for the facial proportions. Control group (CG). Class II dentofacial deformity (DFD II). Class III dentofacial deformity (DFD III)

Furthermore, significant differences were found between the DFD groups. DFD II showed decreased distances for middle face width (tr-tl), lower facial depth (t-gn), right side (tr-chr), and left side (tl-chl). In addition, DFD II presented greater lower facial height (sn-gn) and lip height (sn-sto) than the DFD III group. The clinical relevance of these differences ranged from moderate to large.

### Surface facial proportions

Significant differences (p ≤ 0.05) in facial proportions were observed between groups, except for the proportion measurement between the sides (Table 1). The facial height/facial width ratio (g-gn/tr-tl) was significantly higher in the DDF II group, which also had the lowest upper facial height/lower facial height ratio (g-sn/sn-gn). The DDF III group presented lower middle facial depth/lower facial depth (t-sn/t-gn) and lip height/chin height (sn-sto/sto-gn) ratios compared to the other groups. The effect size of these differences ranged from small to large.

### Maximum bite force

The MBF values were lower in the DFD groups compared to the CG, with moderate clinical relevance. However, no significant difference was found between the sides or between the DFD II and DFD III groups (Table 2).

**Table 2:** Maximum bite force and occlusal contact area among the three groups

**Variables**	**CG (n=25)**	**DFD II (n=20)**	**DFD III (n=32)**	P-Value *	$E_{R}^{2}$
MBF right side (N)	433.3 (219.2) ^a^	156.2 (125.3) ^b^	193.2 (155.5) ^b^	<.0001^*^	0.470
MBF left side (N)	435.4 (189.2) ^a^	181.2 (130.7) ^b^	181.0 (152.4) ^b^	<.0001*	0.405
Total MBF (N)	439.1 (185.8) ^a^	167.4 (140.1) ^b^	194.3 (148.2) ^b^	<.0001*	0.455
Occlusal contact area right side (mm^2^)	107.2 (67.2) ^a^	34.4 (32.0) ^b^	23.2 (22.8) ^b^	<.0001*	0.537
Occlusal contact area left side (mm^2^)	104.0 (51.2) ^a^	36.8 (27.2) ^b^	21.6 (23.2) ^b^	<.0001*	0.603
Total occlusal contact area (mm^2^)	208.0 (112.0) ^a^	77.6 (48.0) ^b^	44.0 (46.8) ^b^	<.0001*	0.604
Asymmetry between sides (%)	5.5 (8.0) ^a^	7.8 (15.7) ^a^	29.2 (23.6) ^b^	0,0001*	0.217

Kruskal-Wallis test*. Median values. Interquartile range (IQR). Superscript letters indicate differences in Dunn’s post-test, and the epsilon squared ( $E_{R}^{2}$ ) represents the effect size. Control group (CG). Class II dentofacial deformity (DFD II). Class III dentofacial deformity (DFD III). Maximum bite force on the right side (MBF right side). Maximum bite force on the left side (MBF left side). Maximum bite force on the right and left sides (total MBF). Occlusal contact area on the right side. Occlusal contact area on the left side. Total occlusal contact area on the right and left sides. Occlusal contact asymmetry between sides

### Occlusal contact area

There was a significant difference between the DFD groups and CG, with a lower occlusal contact area to DFD II and DFD III, although no difference was found between them. The asymmetry between sides was significant in DFD III compared to DFD II and GC (Table 2). The clinical relevance of these findings ranged from moderate to large.

### Relationship between MBF and 3D facial morphology

There was a significant correlation between 3D facial morphology and MBF, with Spearman correlation coefficients ranging from weak to moderate. Generally, the linear distances of middle face width (tr-tl) and middle facial depth (t-sn) showed a positive correlation with MBF, while the facial height/facial width ratio (g-gn/tr-tl) showed a negative correlation with MBF (Table 3). The multiple linear regression analysis identified several predictive variables, including age, gender, and anthropometric measurements, such as chin height (sto-gn), middle face width (tr-tl), lip height (sn-sto), and middle facial depth (t-sn). Furthermore, the proportional measurements most significantly associated with MBF magnitude were facial height/facial width (g-gn/tr-tl) and lip height/chin height (sn-sto/sto-gn) (R2 ranged from 0.328 to 0.382), as shown in Table 4.

**Table 3:** Spearman’s rank correlation coefficients and relevant p-values

**Bite Force (N) vs**	**Spearman Rho**	P-value*
**Linear distances (mm)**		
Total facial height (g-gn)	-0.0710	0.54
Middle face width (tr-tl)	0.4469	<.0001*
Upper facial height (g-sn)	-0.0407	0.73
Lower facial height (sn-gn)	-0.0863	0.46
Lip height (sn-sto)	0.0472	0.68
Chin height (sto-gn)	-0.1794	0.12
Middle facial depth (t-sn)	0.5189	<.0001^*^
Lower facial depth (t-gn)	0.2903	0.0104*
Left side (tl-chl)	0.3561	0.0015*
Right side (tr-tr)	0.3574	0.0014*
**Facial proportions (%)**		
Facial height/facial width (g-gn/tr-tl)	-0.4377	<.0001*
Upper facial height/lower facial height (g-sn/sn-gn)	0.0320	0.78
Middle facial depth/lower facial depth (t-sn/t-gn)	0.1492	0.20
Lip height/chin height (sn-sto/sto-gn)	0.1356	0.24

Spearman’s correlation test (p ≤ 0.05*)

**Table 4:** Multiple regression analysis of linear distances and facial proportions associated with maximum bite force

**R^2^**	**F (P-value)**	**Variables**	**Slope**	P-value*
0.382	8.77 (<.0001)	Age (years)	-8.84	0.003^*^
		Middle face width (mm)	8.10	0.001*
		Chin height (mm)	-12.07	0.002*
		Lip height (mm)	6.12	0.033*
		Middle facial depth (mm)	5.55	0.048*
				
0.328	6.93(<.0001)	Sex (0=F; 1=M)	77.81	0.047*
		Age (years)	-8.39	0.007*
		Facial height/facial width (%)	-10.03	0.004*
		Lip height/chin height (%)	5.89	0.050*
		Middle facial depth/lower facial depth (%)	5.52	0.08

P-value < 0.05* indicates a statistically significant difference

**Caption:** R^2^ = coefficient of determination; F (P-value) = evaluation of the model; Slope = linear regression inclination

## DISCUSSION

In this study, we observed that DFD patients exhibited variations in 3D facial soft tissue dimensions beyond normal limits. The DFD II and DFD III groups showed differences in 3D surface facial measurements, including transversal axis (*x)* discrepancies, such as middle face width (tr-tl), vertical axis *(y)* discrepancies, such as lip height (sn-sto), and anteroposterior axis *(z)* discrepancies, such as lower facial depth (t-gn). These dimensions demonstrated a significant relationship with MBF. However, there were no differences in MBF and occlusal contact area between DFD II and DFD III groups. Nonetheless, we found lower MBF values and occlusal contact area in patients compared to the CG. In addition, the DFD groups presented an increase in the vertical measurement of chin height (sto-gn), and this 3D linear distance had the greatest negative association with MBF.

Conventional anthropometry has been previously used to evaluate facial morphology^(6)^. Three-dimensional laser surface scanning offers advantages over 2D methods, since it is non-ionizing, non-invasive, fast, reliable, and reproducible^(16)^. In this study, we used the FastSCAN^TM^ method, which demonstrated high reliability of anthropometric measurements without systematic errors in both inter-rater and intra-rater analyses, similar to previous findings^(16)^. The linear distances obtained through laser scanning, cone beam computed tomography, and 3D stereo-photogrammetry were found to be reliable and accurate compared to direct anthropometric measurements. Additionally, no differences were observed between the 3D techniques in terms of anthropometric analyses, which are relevant for research and clinical practices^(25)^.

Previous studies have investigated facial dimensions in soft tissue profiles using a 3D approach before orthognathic surgery^(12)^. However, there are fewer studies addressing skeletal Class II malocclusion. Our findings revealed the smallest lower facial depth (t-gn) in the DFD II group, which is consistent with previous 3D results^(26)^ and is associated with retrognathia. Moreover, this group presented increased chin height (sto-gn), lower facial height (sn-gn), and total facial height (g-gn). Preliminary cephalometric analyses have reported an increase in vertical dimensions related to skeletal Class II malocclusion^(11)^, which supports the relationship between adjacent tissues.

In the vertical axis, in Class III skeletal malocclusion, there is a strong correlation between soft and hard tissue lower anterior facial heights using a 2D approach^(6)^. In this study, the DFD III group demonstrated the greatest lower facial depth (t-gn), which has been previously reported^(27)^ and is associated with mandibular prognathism^(28)^. In addition, there was an increase in chin height (sto-gn) in the vertical dimension. Although the DFD III group tended towards a long face, no difference was found in total facial height (g-gn), which is consistent with the results observed in other studies^(12,13)^.

Our results indicate that soft tissue structures adapt to unfavorable skeletal relationships, leading to impairments in the masticatory function. The magnitude of MBF and occlusal contact area were reduced in the DFD groups compared to the CG, which is consistent with similar studies^(2-4)^. Iwase (2006)^(2)^ reported that occlusal contact area is a determining factor for MBF, and in DFD patients, reduced MBF is directly related to the number of occlusal contacts. During presurgical orthodontic treatment, dental decompensation further decreases MBF and the occlusal contact area^(3)^. Additionally, patients with long faces tend to have few and smaller type II fibers, resulting in relatively weak bite forces^(29)^. The MBF values and occlusal contact area did not differ between the DFD II and DFD III groups, which is consistent with previous findings^(4,5)^. However, the asymmetry between sides in the DFD III group might be related to posterior crossbite dental malocclusion, which is sometimes present in Class III DFD.

The relationship between masticatory muscles and facial morphology has been observed before. For instance, masseter thickness was found to be positively correlated with the horizontal dimension of the mandible in adults with mandibular prognathism, while a negative correlation was observed between masseter thickness and the severity of DFD^(30)^. Furthermore, higher MBF values were found to be correlated with thicker masseter muscle and greater face width in healthy subjects^(31)^. In this study, we found a positive association between the transversal dimension of middle face width (tr-tl) and MBF.

In contrast, the vertical dimension of chin height (sto-gn) showed a negative association with MBF. Therefore, our results suggest that the increase in this dimension in the DFD II and DFD III groups could contribute to lower MBF magnitude in these skeletal malocclusions. Individuals with short faces, upright mandible ramus, and acute gonial angle tend to have a greater mechanical advantage for the elevator muscles of the mandible and higher MBF^(9)^. Conversely, individuals with long faces tend to have a reduced biomechanical advantage of the jaw muscles and lower MBF^(9-11)^.

Additionally, we found that maxillomandibular length was not correlated with MBF in DFD patients^(10)^. On the other hand, we observed a positive correlation in the anteroposterior axis (*z*) between middle facial depth (t-sn) and lower facial depth (t-gn) with MBF, which suggests that these linear distances need to be balanced to yield a positive influence on MBF. However, DFD typically exhibit unfavorable maxillomandibular relationships, as reflected in the 3D soft tissue dimensions^(12)^.

In this study, the relationship between form and function became clearer, demonstrating that skeletal DFD affected the dimensions of the facial soft tissue and led to a reduction in the bite force magnitude and occlusal contact area. In clinical practice, this can be reflected in the disharmony of the masticatory muscles and compromised masticatory efficiency in DFD patients, as revealed in previous studies^(2,32)^. These unfavorable conditions lead to a functional adaptation with impairments in the masticatory function.

The limitations of this study include the number of participants in the DFD II group, since most patients seeking treatment at the university hospital have Class III skeletal malocclusion. Additionally, the sample size was based on convenience sampling. Based on these findings, we emphasize the importance of considering 3D soft tissue variations in skeletal malocclusions and their relationship with the masticatory function. Future studies could investigate whether changes in facial morphology after orthognathic surgery influence the improvement process of the masticatory function.

## CONCLUSION

In conclusion, we observed significant impairments in MBF and occlusal contact area in the DFD groups, indicating compromised masticatory function components. The 3D facial soft tissue dimensions also presented variations beyond normal limits in DFD patients. Despite the morphological differences between the two groups, both Class II and Class III DFD groups showed an increase in chin height (sto-gn) in the vertical axis (*y*), which might have contributed to the lower MBF magnitude in both skeletal deformities. Our findings highlight the importance of considering the relationship between 3D facial soft tissue and the MBF in DFD patients, as being relevant for the clinical measurements. Regarding facial morphology, there are also functional problems and not only aesthetic concerns. These can contribute to enhance myofunctional diagnosis and therapeutic planning for the masticatory function, both before and after orthognathic surgery.
